# Supplementary material for: Antiestrogenic Activity and Possible Mode of Action of Certain New Nonsteroidal Coumarin-4-acetamides
Source: Molecules. 2020 Mar 28;25(7):1553. doi: 10.3390/molecules25071553 (PMC7181245; doi:10.3390/molecules25071553)
Supplement: Supplementary file 1 [file molecules-25-01553-s001.pdf]

Elemental analysis of newly obtained compounds **IIIa –h**

Sample Code	M Formula	M.wt g/mol	C%		H%		N%	
			Calc.	Found	Calc.	Found	Calc.	Found
**IIIa**	$C_{17}H_{19}NO_4$	301.34	**67.76**	68.01	**6.36**	6.67	**4.65**	4.50
**IIIb**	$C_{17}H_{19}NO_4$	301.34	**67.76**	67.99	**6.36**	6.35	**4.65**	4.49
**IIIc**	$C_{18}H_{15}NO_4$	309.32	**69.89**	69.57	**4.89**	4.66	**4.53**	4.44
**IIId**	$C_{18}H_{14}NO_4Br$	388.21	**55.69**	55.56	**3.63**	3.21	**3.61**	3.57
**IIIe**	$C_{19}H_{17}NO_6$	355.34	**64.22**	64.10	**4.82**	4.74	**3.94**	3.89
**IIIf**	$C_{18}H_{15}NO_4$	309.32	**69.89**	67.17	**4.89**	4.59	**4.53**	4.22
**IIIg**	$C_{18}H_{14}NO_4Br$	388.21	**55.69**	55.39	**3.63**	4.1	**3.61**	3.60
**IIIh**	$C_{19}H_{17}NO_6$	355.34	**64.22**	65.98	**4.82**	5.10	**3.94**	4.31
